# Editorial: Molecular Mechanisms in Cancer Endocrinology With Clinical Implications

**DOI:** 10.3389/fendo.2022.837705

**Published:** 2022-02-28

**Authors:** Naris Nilubol

**Affiliations:** National Cancer Institute, National Institutes of Health (NIH), Bethesda, MD, United States

**Keywords:** thyroid cancer (THCA), adrenal cancer, neuroendocrine neoplasia, pheochromacytoma, hyperaldosteronism, Cushing syndrome (CS)

Endocrine neoplasms are unique tumors with variable malignant potential and diverse clinical characteristics. In addition to the tumor progression and metastasis seen in other neoplasms, patients with endocrine tumors often present with syndromes related to excessive production of metabolically-active hormones, cytokines, and/or various peptides. The presentations range from asymptomatic patients with abnormal routine laboratory finding such as that seen in primary hyperparathyroidism, or incidentally-discovered adrenal or pancreatic lesions, to catastrophic conditions seen in patients with pheochromocytoma or carcinoid crises. Similarly, tumor size can range from occult or microscopic disease such as in ectopic ACTH-producing tumors or insulinoma to large tumor burden in patients with metastatic neuroendocrine tumors or adrenocortical cancer. The improved understanding of tumor biology and underlying molecular alterations associated with tumor initiation and progression in endocrine cancers has increasingly been translated into clinical use resulting in more accurate diagnostic tests, risk-stratification, novel and sensitive theranostic imaging studies, and targeted systemic therapy in several endocrine cancers. In this Research Topic, the authors discussed the key molecular alterations that have important clinical implications in thyroid cancer, familial parathyroid tumor syndromes, functioning adrenocortical neoplasms, pheochromocytoma, and paraganglioma, small bowel, and pancreatic neuroendocrine tumors.

To address the clinical challenge in accurate risk-stratification and managing thyroid nodules with indeterminate cytology, adjunct molecular testing such as Afirma and ThyroSeq are commercially available. Khan and Zeiger discussed the diagnostic performance in cytologically indeterminate thyroid nodules based on the data from original articles and from “real-world” studies. More importantly, the data on the impact of these molecular testing on surgical practice, decision-making process, and cost-effectiveness. The insight into molecular alterations in thyroid cancer progression has led to the incorporation of biomarkers associated with aggressive thyroid cancer into molecular testing. Telomerase reverse transcriptase (TERT) dysregulation is one of the key alterations seen in several cancers, including poorly-differentiated and anaplastic thyroid cancer. McKelvey et al. comprehensively review the regulation of TERT, focusing on *TERT* promoter mutations, epigenetic changes at the promoter, and the relevant clinical significance. Because radioiodine-refractory thyroid cancer continues to be the leading cause of thyroid caner-related mortality, Fullmer et al., discussed the treatment options, which are small molecule inhibitors of pathogenic signaling pathways in radioiodine-refractory thyroid cancers. Similarly, Okafor et al. reviewed the RET and RAS signaling pathway in the medullary and discussed treatment strategies.

Adrenocortical tumors have diverse clinical presentations and unique molecular alterations specific to subtypes of these tumors. Kamilaris et al. summarized the pathogenic variants in genes encoding ion transporters in aldosterone-producing tumors, such as *KCNJ5, CACNA1D, ATP1A1, ATP2B3* somatic variants. Unlike aldosterone-producing tumors, cortisol-producing adrenal tumors have different types of somatic and germline pathogenic variants. Some of the dysregulated pathways in cortisol-producing adrenal tumors can be seen in adrenocortical cancer. We comprehensively reviewed clinical features and challenges in managing patients with adrenocortical cancer because of the lack of effective systemic therapy. We discussed the key molecular alterations and provided a summary of the previously tested treatment regimens in patients with ACC that were mostly ineffective. The current clinical trials that are actively enrolling patients with ACC were included.

This Research Topic includes several articles that focus on unique aspects of neuroendocrine tumors. Chatani et al. summarized the key molecular alterations in pancreatic neuroendocrine tumors such as germline and somatic mutations of *MEN1*, *DAXX/ATRX*, genes in PI3K/AKT/mTOR, and hypoxic response signaling pathways. The review discussed the unique molecular mechanisms in pancreatic neuroendocrine *carcinoma* with additional *RB* and *TP53* mutations. The article included the therapeutic options that target these dysregulated pathways. Lim and Pommier summarized molecular characteristics, clinical features, and management of familial small bowel neuroendocrine tumors. The authors reviewed molecular features, clinical presentations, and management of sporadic and familial small bowel neuroendocrine tumors. Pheochromocytoma and paraganglioma are rare neuroendocrine tumors that can secret catecholamines. Patel et al. presented a unique aspect of these tumors that overexpress somatostatin receptors, similar to other neuroendocrine tumors. The authors summarized the clinical implications of somatostatin receptor-targeted diagnostic modalities and various therapeutic approaches.

Hyperparathyroidism remains a common endocrine disorder that frequently requires accurate imaging studies to guide the extent of surgical resection. Given the recent progress in parathyroid imaging techniques, Morris et al. reviewed various imaging modalities and provided a summary of the test performance. In addition to the conventional localizing imaging studies such as ultrasonography, sestamibi scan, and 4-D CT scan, the authors discussed the performance of newer radioisotope scintigraphy such as 18F-Fluorocholine flurpiridaz. Blau and Simonds summarized the current knowledge about etiology, clinical characteristics of familial hyperparathyroidism caused by mutations in *MEN1, CDC73, CASR*, and *GCM2* genes.

In summary, this Research Topic includes the review articles focusing on molecular mechanisms of endocrine neoplasms derived from thyroid, parathyroid, adrenal glands, and neuroendocrine cells in the pancreas and small bowel. All articles highlight the clinical relevance and practical points that are useful to a broad range of readers.

## Author Contributions

The author confirms being the sole contributor of this work and has approved it for publication.

## Funding

This work was supported by NIH Intramural Research Program, grant #ZIA BC 011286.

## Conflict of Interest

The author declares that the research was conducted in the absence of any commercial or financial relationships that could be construed as a potential conflict of interest.

## Publisher’s Note

All claims expressed in this article are solely those of the authors and do not necessarily represent those of their affiliated organizations, or those of the publisher, the editors and the reviewers. Any product that may be evaluated in this article, or claim that may be made by its manufacturer, is not guaranteed or endorsed by the publisher.

